# Chronic subdural hematoma leading to fatal cavernous sinus thrombosis

**DOI:** 10.4103/0974-2700.44684

**Published:** 2009

**Authors:** Sunil Kumar, A P Jain, S Jain, S K Kale

**Affiliations:** Department of Medicine, Mahatma Gandhi Institute of Medical Sciences, Sewagram, Wardha, (Maharashtra), India; 1 Department of ENT, Mahatma Gandhi Institute of Medical Sciences, Sewagram, Wardha, (Maharashtra), India; 2 Department of Radiodiagnosis, Mahatma Gandhi Institute of Medical Sciences, Sewagram, Wardha, (Maharashtra), India

**Keywords:** Cavernous sinus thrombosis, chronic, subdural hematoma

## Abstract

Presented is a case of cavernous sinus thrombosis in a young female with fatal outcome. There were not any septic focus, no history of head trauma, no relation with pregnancy. Computed tomography scan of brain showed chronic subdural hematoma. An attempt is made to correlate the aetiopathology with the clinical features of this rare case presentation.

## INTRODUCTION

A subdural hematoma (SDH) is a form of traumatic brain injury in which blood collects between the dura and the arachnoid. Unlike in epidural hematomas, which are usually caused by tears in arteries, subdural bleeding usually results from tears in veins that cross the subdural space. We present a case report of a young adult female with cavernous sinus thrombosis associated with old SDH with resultant poor outcome.

## CASE REPORT

A 30-year-old female presented to the emergency department of the hospital with diffuse headache and bilateral proptosis, which was associated with vomiting. There was no ear discharge, cough, or burning micturition. There was no history of trauma, or seizures. She had no past history of convulsions or tuberculosis. She had no history of sickle cell disease, hypertension, and diabetes. She was P2L2A0 and the previous deliveries were normal without any complicating events.

On examination, she was conscious, but drowsy with low-grade fever.glasgow coma scale was 9 at the time of admission. She was pale and anicteric. Her pulse was 120/min and regular. Her blood pressure on admission was 130/90 mmHg in left upper limb supine position. Main neurological finding was left third nerve palsy with papilloedema.

**Figure 1:**
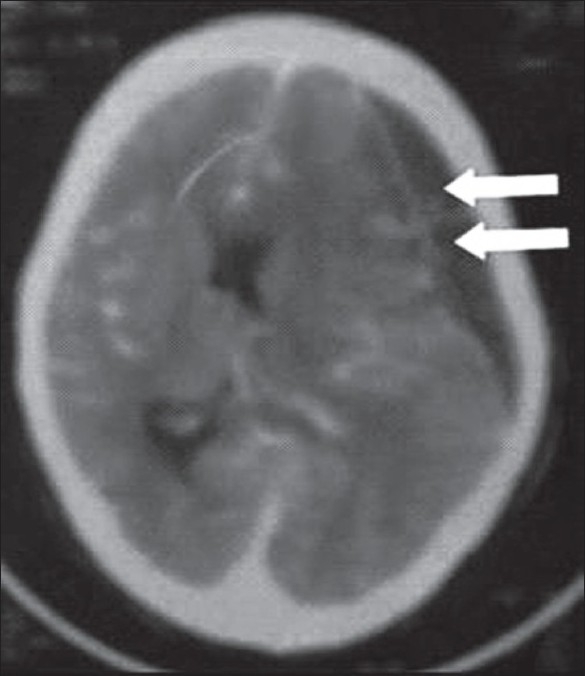
CT Image showing bilateral ophthalmic vein thrombosis with cavernous sinus thrombosis

**Figure 2:**
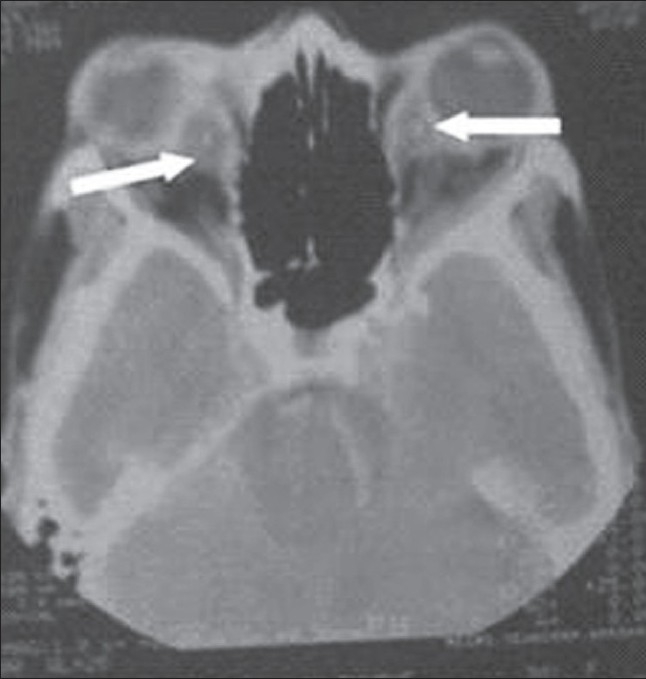
CT Image showing subdural hematoma in same patient

Local examination of eyes showed bilateral proptosis which was associated with chemosis and corneal haziness. Other systemic examination was unremarkable. Her hemoglobin was 8.5 gm/dl, hematocrit was 26%, total leukocyte counts were 15,000 per cubic mm. Blood culture was sterile. Platelets were normal. There was no sickle cell on peripheral smear. There was no deficiency of protein C or S. Antiphospholipids antibody was normal. Her chest film was normal. Patient underwent plain and post-contrast computed tomography (CT) head scan which showed bilateral superior ophthalmic vein thrombosis extending to the thrombosed cavernous sinus [Figure 1]. There was also right subdural hematoma (appears to be chronic) with midline shift [Figure 2]. There was no sign of cerebritis (heterogeneous enhancing area in adjoining hypodense area) in adjacent cerebral parenchyma, so chances of empyema were less likely. On the basis of this CT scan findings and clinical feature, we treated the patients with higher antibiotics in the form of combination of sulbactum and ceftrixone intravenously twice a day and corticosteroids. Heparin was started in view of high-risk benefit ratio of cavernous sinus thrombosis and subdural hematoma. Neurosurgical consultation was also taken, hematoma (there were no evidence of pus) was evacuated. In spite of all, patient did not show any improvement and died subsequently. We did not send her for autopsy due to resistance by her relatives.

## DISCUSSION

Cavernous sinus thrombosis (CST) initially described by Bright in 1831 are a complication of epidural and subdural infections.[1] Exact incidence of CST is still under debate because of scarcity of scientifically planned epidemiological studies in the available literature. Hospital data has been utilized to determine its prevalence in the community.[2] In Indian subcontinent, post-puerperal thrombosis being the commonest, clinical picture usually comprises a young premenopausal female, who 7–10 days after normal delivery. CST may be due to infection of the central face or paranasal inuses, bacteremia, trauma, and infections of the ear or maxillary teeth. Though there was no septic focus either on face, eye or nose in our patient. In CT imaging, there was superior ophthalamic vein thrombosis leading to cavernous sinus thrombosis. Signs of CST may be periorbital edema (usually the earliest finding), chemosis (due to occlusion of the ophthalmic veins), lateral gaze palsy (cranial nerve VI), ptosis, mydriasis, and eye muscle weakness from cranial nerve III dysfunction. Manifestations of increased retrobulbar pressure may be as exophthalmos and ophthalmoplegia. Appearance of signs and symptoms in the contralateral eye is diagnostic of CST, although the process may remain confined to one eye. In this case bilateral proptosis, chemosis, and bilateral pappiloedema were the main physical finding. Apart from this on CT image of the brain had shown subdural hematoma which appears to be chronic in nature. Though there was no history of any trivial trauma, or any risk factor, we are correlating this cavernous sinus thrombosis as a complication of chronic subdural hematoma.

In this patient, on contrast enhancing computed tomography head scan there is evidence of nonenhancing extraxial crescent shaped hypodense area in between the skull vault and brain surface in right frontoperital region causing midline shift and compression over ipsilateral ventricle suggestive of chronic subdural hematoma. Often, a chronic SDH will appear as a heterogeneously dense lesion indicative of recurrent bleeding with a fluid level between the acute (hyperdense) and chronic (hypodense) components of the hematoma.[3]

Intracranial bleeding in venous thrombosis is a consequence of increased venous and capillary pressure and thus occurs more frequently than in arterial thrombotic disease. Erosion of the cavernous sinus thrombosis is the proposed mechanism for development of the SDH[4] (should be acute or subacute). There is also the possibility of occurrence of subdural hematoma due to occlusion of cerebral vein. The occlusion of the cerebral veins can cause localized edema of the brain and venous infarction. Pathological examination shows enlarged, swollen veins, edema, ischemic neuronal damage, and petechial hemorrhages. The latter can merge and become large hematomas.[5] In this case, etiology of thrombosis was not known though infection was the possibility because leucocyte counts were raised. Few case reports are available of subdural hematoma in a case of ruptured[6,7] or unruptured[2] thrombosed intracavernous carotid artery aneurysm. Search were made on PUBMED, medlars and Indian medlars there was no such case report published regarding subdural hematoma associated with cavernous sinus thrombosis.

## CONCLUSION

Sinus thrombosis may present to the physician in a number of guises. Diagnosis can be confirmed by computed tomography imaging in most cases. Cavernous sinus thrombosis is a rare complication of subdural hematoma. Though we exactly could not correlate the etiopathogenesis of this case presentation, early recognition of the condition and instigation of appropriate therapy could probably reduce mortality and morbidity. However, more case studies and post mortem examinations are required to prove this hypothesis.

## References

[1] Sharma R (2006). Cavernous sinus thrombosis. Emedicine.

[2] Prakash C, Bansal BC (2005). Cerebral venous thrombosis. J Indian Acad Clin Med.

[3] Sinson GP, Reiter GT (2002). Emedicine: subdural hematoma.

[4] Triantafyllopoulou A, Beaumont A, Ulatowski J, Tamargo RJ, Varelas PN (2006). Acute subdural hematoma caused by an unruptured, thrombosed giant intracavernous aneurysm: Case report. Neurocrit Care.

[5] Stam J (2005). Thrombosis of the cerebral veins and sinuses. N Engl J Med.

[6] Barr HW, Blackwood W, Meadows SP (1971). Intra cavernous carotid aneurysms. Brain.

[7] Hodes JE, Fletcher WA, Goodman DF, Hoyt WF (1988). Rupture of cavernous artery aneurysm causing subdural hematoma and death. J Neurosurg.

